# 5-[(*E*)-2-Fluoro­benzyl­idene]-8-(2-fluoro­phen­yl)-2-hy­droxy-10-methyl-3,10-di­aza­hexa­cyclo­[10.7.1.1^3,7^.0^2,11^.0^7,11^.0^16,20^]henicosa-1(20),12,14,16,18-pentaen-6-one

**DOI:** 10.1107/S1600536810052815

**Published:** 2010-12-24

**Authors:** Raju Suresh Kumar, Hasnah Osman, Chin Sing Yeap, Hoong-Kun Fun

**Affiliations:** a School of Chemical Sciences, Universiti Sains Malaysia, 11800 USM, Penang, Malaysia; b X-ray Crystallography Unit, School of Physics, Universiti Sains Malaysia, 11800 USM, Penang, Malaysia

## Abstract

In the title compound, C_33_H_26_F_2_N_2_O_2_, the piperidone ring adopts a half-chair conformation and the pyrrolidine rings adopt half-chair and envelope conformations. The two benzene rings make dihedral angles of 29.58 (5) and 76.33 (5)° with the mean plane of the 1,2-dihydro­acenaphthyl­ene unit. An intra­molecular O—H⋯N hydrogen bond helps to stabilize the mol­ecular structure. In the crystal, inter­molecular C—H⋯F hydrogen bonds link the mol­ecules into [010] chains. Weak C—H⋯π inter­actions are also observed.

## Related literature

For general background to and the biological activity of heterocyclic compounds, see: Tsuge & Kanemasa (1989 ▶); Grigg & Sridharan (1993 ▶); Daly *et al.* (1986 ▶); Waldmann (1995 ▶). For the synthesis, see: Kumar *et al.* (2010 ▶). For ring conformations, see Cremer & Pople (1975 ▶). For the stability of the temperature controller used in the data collection, see: Cosier & Glazer (1986 ▶).
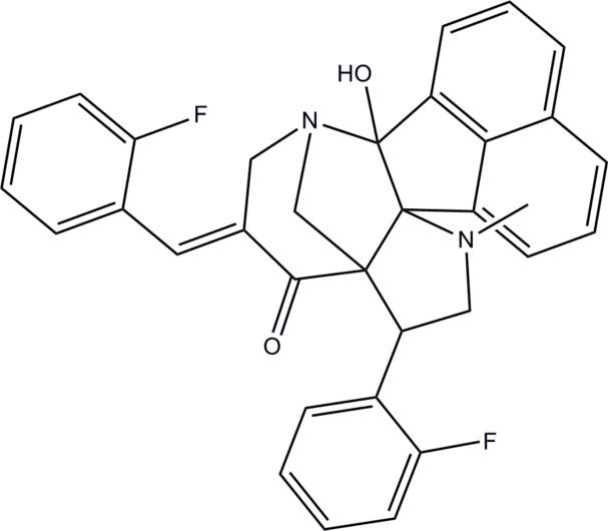


## Experimental

### 

#### Crystal data

C_33_H_26_F_2_N_2_O_2_
*M* *_r_* = 520.56
Monoclinic, 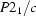

*a* = 16.664 (2) Å
*b* = 9.7226 (11) Å
*c* = 15.507 (2) Å
β = 96.447 (2)°
*V* = 2496.5 (5) Å^3^
*Z* = 4
Mo *K*α radiation
μ = 0.10 mm^−1^
*T* = 100 K
0.49 × 0.32 × 0.13 mm

#### Data collection

Bruker APEXII DUO CCD diffractometer
Absorption correction: multi-scan (*SADABS*; Bruker, 2009 ▶) *T*_min_ = 0.955, *T*_max_ = 0.988
31956 measured reflections
10068 independent reflections
7622 reflections with *I* > 2σ(*I*)
*R*_int_ = 0.040

#### Refinement

*R*[*F* ^2^ > 2σ(*F* ^2^)] = 0.052
*wR*(*F* ^2^) = 0.181
*S* = 1.09
10068 reflections
357 parameters
H atoms treated by a mixture of independent and constrained refinement
Δρ_max_ = 0.50 e Å^−3^
Δρ_min_ = −0.34 e Å^−3^

### 

Data collection: *APEX2* (Bruker, 2009 ▶); cell refinement: *SAINT* (Bruker, 2009 ▶); data reduction: *SAINT*; program(s) used to solve structure: *SHELXTL* (Sheldrick, 2008 ▶); program(s) used to refine structure: *SHELXTL*; molecular graphics: *SHELXTL*; software used to prepare material for publication: *SHELXTL* and *PLATON* (Spek, 2009 ▶).

## Supplementary Material

Crystal structure: contains datablocks global, I. DOI: 10.1107/S1600536810052815/hb5771sup1.cif

Structure factors: contains datablocks I. DOI: 10.1107/S1600536810052815/hb5771Isup2.hkl

Additional supplementary materials:  crystallographic information; 3D view; checkCIF report

## Figures and Tables

**Table 1:** Hydrogen-bond geometry (Å, °) *Cg*1 and *Cg*2 are the centroids of the C14–C18/C23 and C1–C6 rings, respectively.

*D*—H⋯*A*	*D*—H	H⋯*A*	*D*⋯*A*	*D*—H⋯*A*
O2—H1*O*2⋯N2	0.88 (3)	2.06 (3)	2.6786 (13)	127 (2)
C10—H10*A*⋯F1^i^	0.97	2.37	3.3135 (14)	163
C30—H30*A*⋯F2^ii^	0.93	2.45	3.1525 (17)	132
C5—H5*A*⋯*Cg*1^iii^	0.93	2.94	3.6110 (14)	131
C33—H33*C*⋯*Cg*2^iv^	0.96	2.93	3.7253 (15)	141

Symmetry codes: (i) 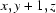; (ii) 
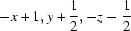
; (iii) 
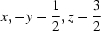
; (iv) 
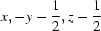
.

**Fig. 1.**
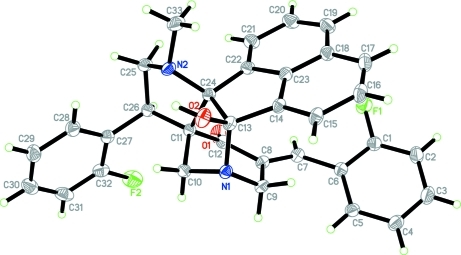
The molecular structure of the title compound with 50% probability ellipsoids for non-H atoms.

**Fig. 2.**
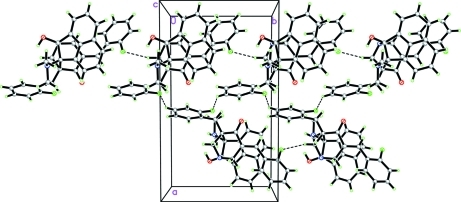
The crystal packing of title compound, viewed down the c axis, showing the molecules linked into chains along the b axis. Intermolecular hydrogen bonds are shown as dashed lines.

## supplementary crystallographic information

### Comment

Due to their varying biological activities, heterocyclic compounds with fused five- and six-membered rings occupy an important place among organic compounds. 1,3-Dipolar cycloadditions are efficient methods for the construction of heterocyclic rings (Tsuge & Kanemasa, 1989; Grigg & Sridharan, 1993). In particular, the chemistry of azomethine ylides gained significance as it serves as a facile route for the construction of nitrogen-containing five-membered heterocycles which constitute the central skeleton of numerous natural products (Daly *et al.*, 1986; Waldmann, 1995). Taking into account the importance of aforesaid heterocycles, we have undertaken the X-ray diffraction study of the title compound and the results are presented here.

The molecular structure of the title compound is shown in Fig. 1. The 4-piperidone ring (C8—C9/N1/C10—C12) adopts a half-chair conformation, with puckering parameters Q = 0.6127 (11) Å, θ = 142.74 (10)° and φ = 119.46 (17)° (Cremer & Pople, 1975). The two fused pyrrolidine rings with atom sequences N1/C10/C11/C24/C13 and N2/C24/C11/C26/C25, adopt a half-chair (twist on N1–C10) and an envelope (flap on atom N2) conformations, respectively. The puckering parameters are Q = 0.4498 (11) Å, φ = 206.94 (14)° for the N1/C10/C11/C24/C13 pyrrolidine ring and Q = 0.4187 (11) Å, φ = 359.86 (16)° for the N2/C24/C11/C26/C25 pyrrolidine ring. The two benzene rings (C1–C6 and C27–C32) make dihedral angles of 29.58 (5) and 76.33 (5)°, respectively with the mean plane of 1,2-dihydroacenaphthylene (C13–C24). The geometric parameters are consistent to those observed in a closely related structure (Kumar *et al.*, 2010). An intramolecular O2—H1O2···N2 hydrogen bond stabilize the molecular structure.

In the crystal structure, intermolecular C10—H10A···F1 and C20—H30A···F2 hydrogen bonds (Table 1) link the molecules into chains propagating along the [010] direction (Fig. 2). Weak intermolecular C—H···π interactions (Table 1) are also observed.

### Experimental

The title compound was synthesized according to the previous procedure described by us (Kumar *et al.*, 2010) and was recrystallized from ethyl acetate to afford pale yellow plates.

### Refinement

The O bound hydrogen atom was located from the difference Fourier map and was refined freely. The rest of hydrogen atoms were positioned geometrically [C–H = 0.93–0.97 Å] and refined using a riding model, with *U*_iso_(H) = 1.2 or 1.5*U*_eq_(C). A rotating-group model was applied for methyl group.

### Crystal data

**Table:**

C_33_H_26_F_2_N_2_O_2_	*F*(000) = 1088
*M**_r_* = 520.56	*D*_x_ = 1.385 Mg m^−^^3^
Monoclinic, *P*2_1_/*c*	Mo *K*α radiation, λ = 0.71073 Å
Hall symbol: -P 2ybc	Cell parameters from 7762 reflections
*a* = 16.664 (2) Å	θ = 2.8–33.8°
*b* = 9.7226 (11) Å	µ = 0.10 mm^−^^1^
*c* = 15.507 (2) Å	*T* = 100 K
β = 96.447 (2)°	Plate, yellow
*V* = 2496.5 (5) Å^3^	0.49 × 0.32 × 0.13 mm
*Z* = 4	

### Data collection

**Table:**

Bruker APEXII DUO CCD diffractometer	10068 independent reflections
Radiation source: fine-focus sealed tube	7622 reflections with *I* > 2σ(*I*)
graphite	*R*_int_ = 0.040
φ and ω scans	θ_max_ = 34.0°, θ_min_ = 2.8°
Absorption correction: multi-scan (*SADABS*; Bruker, 2009)	*h* = −25→26
*T*_min_ = 0.955, *T*_max_ = 0.988	*k* = −15→15
31956 measured reflections	*l* = −24→22

### Refinement

**Table:**

Refinement on *F*^2^	Primary atom site location: structure-invariant direct methods
Least-squares matrix: full	Secondary atom site location: difference Fourier map
*R*[*F*^2^ > 2σ(*F*^2^)] = 0.052	Hydrogen site location: inferred from neighbouring sites
*wR*(*F*^2^) = 0.181	H atoms treated by a mixture of independent and constrained refinement
*S* = 1.09	*w* = 1/[σ^2^(*F*_o_^2^) + (0.1093*P*)^2^ + 0.1256*P*] where *P* = (*F*_o_^2^ + 2*F*_c_^2^)/3
10068 reflections	(Δ/σ)_max_ < 0.001
357 parameters	Δρ_max_ = 0.50 e Å^−^^3^
0 restraints	Δρ_min_ = −0.34 e Å^−^^3^

### Special details

**Table:**

Experimental. The crystal was placed in the cold stream of an Oxford Cryosystems Cobra open-flow nitrogen cryostat (Cosier & Glazer, 1986) operating at 100.0 (1) K.
Geometry. All e.s.d.'s (except the e.s.d. in the dihedral angle between two l.s. planes) are estimated using the full covariance matrix. The cell e.s.d.'s are taken into account individually in the estimation of e.s.d.'s in distances, angles and torsion angles; correlations between e.s.d.'s in cell parameters are only used when they are defined by crystal symmetry. An approximate (isotropic) treatment of cell e.s.d.'s is used for estimating e.s.d.'s involving l.s. planes.
Refinement. Refinement of *F*^2^ against ALL reflections. The weighted *R*-factor *wR* and goodness of fit *S* are based on *F*^2^, conventional *R*-factors *R* are based on *F*, with *F* set to zero for negative *F*^2^. The threshold expression of *F*^2^ > σ(*F*^2^) is used only for calculating *R*-factors(gt) *etc*. and is not relevant to the choice of reflections for refinement. *R*-factors based on *F*^2^ are statistically about twice as large as those based on *F*, and *R*- factors based on ALL data will be even larger.

### Fractional atomic coordinates and isotropic or equivalent isotropic displacement parameters (Å^2^)

**Table:**

	*x*	*y*	*z*	*U*_iso_*/*U*_eq_	
F1	0.22287 (5)	−0.09340 (8)	−0.08747 (6)	0.03048 (18)	
F2	0.45096 (6)	0.58239 (9)	−0.20151 (6)	0.0358 (2)	
O1	0.39107 (5)	0.30091 (10)	−0.12913 (7)	0.02658 (19)	
O2	0.15163 (5)	0.61493 (9)	0.00816 (6)	0.02169 (17)	
N1	0.17809 (5)	0.51233 (9)	−0.12231 (6)	0.01635 (17)	
N2	0.30632 (5)	0.56856 (10)	0.06815 (6)	0.01923 (18)	
C1	0.16676 (7)	−0.05834 (12)	−0.15376 (8)	0.0215 (2)	
C2	0.10676 (7)	−0.15156 (13)	−0.17951 (10)	0.0271 (2)	
H2A	0.1053	−0.2373	−0.1532	0.033*	
C3	0.04838 (8)	−0.11319 (14)	−0.24619 (10)	0.0304 (3)	
H3A	0.0073	−0.1744	−0.2653	0.037*	
C4	0.05071 (8)	0.01535 (14)	−0.28457 (9)	0.0268 (2)	
H4A	0.0102	0.0412	−0.3277	0.032*	
C5	0.11349 (7)	0.10543 (12)	−0.25857 (8)	0.0211 (2)	
H5A	0.1156	0.1903	−0.2859	0.025*	
C6	0.17377 (7)	0.07036 (11)	−0.19167 (7)	0.0185 (2)	
C7	0.24461 (7)	0.15535 (12)	−0.16437 (8)	0.0194 (2)	
H7A	0.2930	0.1089	−0.1499	0.023*	
C8	0.24723 (6)	0.29276 (11)	−0.15795 (7)	0.01732 (19)	
C9	0.17378 (6)	0.38537 (11)	−0.17402 (7)	0.01779 (19)	
H9A	0.1664	0.4099	−0.2350	0.021*	
H9B	0.1265	0.3338	−0.1617	0.021*	
C10	0.25581 (6)	0.57891 (11)	−0.13048 (7)	0.01756 (19)	
H10A	0.2561	0.6728	−0.1093	0.021*	
H10B	0.2673	0.5795	−0.1904	0.021*	
C11	0.31781 (6)	0.48950 (11)	−0.07365 (7)	0.01573 (18)	
C12	0.32628 (6)	0.35540 (11)	−0.12233 (7)	0.01816 (19)	
C13	0.17937 (6)	0.49274 (11)	−0.02730 (7)	0.01591 (18)	
C14	0.13397 (6)	0.36810 (11)	−0.00054 (7)	0.01704 (19)	
C15	0.05339 (6)	0.33647 (12)	−0.00943 (8)	0.0209 (2)	
H15A	0.0154	0.3981	−0.0354	0.025*	
C16	0.02922 (7)	0.20736 (13)	0.02195 (9)	0.0252 (2)	
H16A	−0.0255	0.1863	0.0174	0.030*	
C17	0.08376 (8)	0.11258 (13)	0.05878 (9)	0.0253 (2)	
H17A	0.0657	0.0289	0.0782	0.030*	
C18	0.16772 (7)	0.14199 (12)	0.06725 (8)	0.0209 (2)	
C19	0.23165 (8)	0.05522 (13)	0.10249 (8)	0.0255 (2)	
H19A	0.2201	−0.0317	0.1228	0.031*	
C20	0.30997 (8)	0.09849 (13)	0.10674 (9)	0.0266 (2)	
H20A	0.3508	0.0400	0.1305	0.032*	
C21	0.33118 (7)	0.22989 (13)	0.07610 (8)	0.0237 (2)	
H21A	0.3850	0.2563	0.0788	0.028*	
C22	0.27098 (6)	0.31696 (12)	0.04254 (7)	0.01815 (19)	
C23	0.19010 (6)	0.27189 (11)	0.03808 (7)	0.01777 (19)	
C24	0.27181 (6)	0.46278 (11)	0.00720 (7)	0.01602 (18)	
C25	0.39317 (6)	0.56622 (13)	0.06012 (7)	0.0211 (2)	
H25A	0.4186	0.4866	0.0894	0.025*	
H25B	0.4192	0.6488	0.0847	0.025*	
C26	0.39858 (6)	0.55880 (11)	−0.03787 (7)	0.01791 (19)	
H26A	0.4428	0.4965	−0.0478	0.021*	
C27	0.41441 (6)	0.69660 (12)	−0.07821 (7)	0.01820 (19)	
C28	0.40392 (7)	0.82279 (13)	−0.03838 (8)	0.0238 (2)	
H28A	0.3833	0.8246	0.0149	0.029*	
C29	0.42350 (8)	0.94592 (14)	−0.07619 (10)	0.0278 (3)	
H29A	0.4157	1.0286	−0.0482	0.033*	
C30	0.45462 (7)	0.94638 (15)	−0.15531 (10)	0.0296 (3)	
H30A	0.4684	1.0291	−0.1799	0.036*	
C31	0.46512 (8)	0.82381 (15)	−0.19762 (9)	0.0294 (3)	
H31A	0.4863	0.8222	−0.2506	0.035*	
C32	0.44319 (7)	0.70319 (13)	−0.15875 (8)	0.0231 (2)	
C33	0.28877 (8)	0.55308 (15)	0.15801 (8)	0.0275 (3)	
H33A	0.3097	0.6309	0.1914	0.041*	
H33B	0.3137	0.4705	0.1821	0.041*	
H33C	0.2314	0.5477	0.1594	0.041*	
H1O2	0.1965 (16)	0.658 (3)	0.0285 (17)	0.073 (8)*	

### Atomic displacement parameters (Å^2^)

**Table:**

	*U*^11^	*U*^22^	*U*^33^	*U*^12^	*U*^13^	*U*^23^
F1	0.0295 (4)	0.0244 (4)	0.0345 (4)	0.0015 (3)	−0.0100 (3)	0.0043 (3)
F2	0.0468 (5)	0.0372 (5)	0.0248 (4)	0.0078 (4)	0.0106 (4)	−0.0036 (3)
O1	0.0161 (3)	0.0257 (4)	0.0385 (5)	0.0032 (3)	0.0052 (3)	−0.0037 (4)
O2	0.0172 (3)	0.0197 (4)	0.0282 (4)	0.0009 (3)	0.0027 (3)	−0.0074 (3)
N1	0.0141 (4)	0.0168 (4)	0.0177 (4)	−0.0003 (3)	−0.0001 (3)	−0.0007 (3)
N2	0.0157 (4)	0.0266 (5)	0.0151 (4)	−0.0052 (3)	0.0007 (3)	−0.0020 (3)
C1	0.0195 (5)	0.0209 (5)	0.0236 (5)	0.0016 (4)	0.0003 (4)	0.0011 (4)
C2	0.0230 (5)	0.0229 (5)	0.0349 (6)	−0.0030 (4)	0.0009 (5)	0.0042 (5)
C3	0.0230 (5)	0.0280 (6)	0.0387 (7)	−0.0069 (4)	−0.0039 (5)	0.0018 (5)
C4	0.0242 (5)	0.0264 (6)	0.0280 (6)	−0.0010 (4)	−0.0049 (5)	−0.0002 (5)
C5	0.0237 (5)	0.0203 (5)	0.0188 (5)	0.0000 (4)	−0.0001 (4)	−0.0005 (4)
C6	0.0186 (4)	0.0185 (4)	0.0187 (5)	0.0006 (3)	0.0029 (4)	−0.0022 (4)
C7	0.0177 (4)	0.0198 (5)	0.0208 (5)	0.0012 (4)	0.0024 (4)	−0.0020 (4)
C8	0.0152 (4)	0.0202 (5)	0.0166 (4)	0.0011 (3)	0.0018 (3)	−0.0015 (4)
C9	0.0160 (4)	0.0192 (4)	0.0174 (4)	0.0003 (3)	−0.0015 (3)	−0.0020 (4)
C10	0.0142 (4)	0.0186 (4)	0.0195 (5)	−0.0003 (3)	0.0000 (3)	0.0024 (4)
C11	0.0121 (4)	0.0184 (4)	0.0165 (4)	−0.0005 (3)	0.0010 (3)	0.0014 (4)
C12	0.0147 (4)	0.0203 (5)	0.0196 (5)	0.0007 (3)	0.0025 (3)	0.0013 (4)
C13	0.0125 (4)	0.0170 (4)	0.0182 (4)	0.0008 (3)	0.0018 (3)	−0.0022 (4)
C14	0.0139 (4)	0.0195 (4)	0.0179 (4)	−0.0017 (3)	0.0025 (3)	−0.0032 (4)
C15	0.0148 (4)	0.0234 (5)	0.0248 (5)	−0.0025 (4)	0.0034 (4)	−0.0051 (4)
C16	0.0191 (5)	0.0277 (6)	0.0295 (6)	−0.0079 (4)	0.0057 (4)	−0.0056 (5)
C17	0.0255 (5)	0.0233 (5)	0.0279 (6)	−0.0082 (4)	0.0065 (4)	−0.0026 (5)
C18	0.0235 (5)	0.0200 (5)	0.0197 (5)	−0.0035 (4)	0.0050 (4)	−0.0020 (4)
C19	0.0329 (6)	0.0209 (5)	0.0230 (5)	−0.0004 (4)	0.0042 (5)	0.0031 (4)
C20	0.0276 (6)	0.0252 (5)	0.0263 (6)	0.0047 (4)	−0.0002 (5)	0.0068 (5)
C21	0.0185 (5)	0.0276 (6)	0.0246 (5)	0.0020 (4)	0.0000 (4)	0.0056 (5)
C22	0.0153 (4)	0.0218 (5)	0.0173 (4)	−0.0001 (3)	0.0014 (3)	0.0016 (4)
C23	0.0163 (4)	0.0205 (5)	0.0168 (4)	−0.0012 (3)	0.0033 (3)	−0.0009 (4)
C24	0.0120 (4)	0.0203 (4)	0.0156 (4)	−0.0013 (3)	0.0009 (3)	0.0003 (4)
C25	0.0145 (4)	0.0301 (5)	0.0181 (5)	−0.0052 (4)	−0.0013 (4)	0.0030 (4)
C26	0.0127 (4)	0.0218 (5)	0.0190 (5)	−0.0014 (3)	0.0007 (3)	0.0014 (4)
C27	0.0118 (4)	0.0239 (5)	0.0188 (5)	−0.0031 (3)	0.0011 (3)	0.0011 (4)
C28	0.0221 (5)	0.0257 (5)	0.0237 (5)	−0.0047 (4)	0.0036 (4)	−0.0003 (4)
C29	0.0235 (5)	0.0241 (5)	0.0347 (7)	−0.0054 (4)	−0.0025 (5)	0.0027 (5)
C30	0.0213 (5)	0.0328 (6)	0.0335 (7)	−0.0050 (4)	−0.0020 (5)	0.0127 (5)
C31	0.0229 (5)	0.0418 (7)	0.0237 (5)	−0.0011 (5)	0.0038 (4)	0.0117 (5)
C32	0.0191 (5)	0.0311 (6)	0.0189 (5)	0.0015 (4)	0.0017 (4)	0.0015 (4)
C33	0.0254 (5)	0.0406 (7)	0.0168 (5)	−0.0087 (5)	0.0034 (4)	−0.0035 (5)

### Geometric parameters (Å, °)

**Table:**

F1—C1	1.3533 (14)	C14—C15	1.3694 (14)
F2—C32	1.3620 (15)	C14—C23	1.4081 (15)
O1—C12	1.2177 (13)	C15—C16	1.4209 (17)
O2—C13	1.4088 (13)	C15—H15A	0.9300
O2—H1O2	0.89 (3)	C16—C17	1.3730 (19)
N1—C10	1.4660 (13)	C16—H16A	0.9300
N1—C9	1.4694 (14)	C17—C18	1.4196 (16)
N1—C13	1.4833 (14)	C17—H17A	0.9300
N2—C33	1.4633 (16)	C18—C23	1.4059 (16)
N2—C25	1.4668 (14)	C18—C19	1.4193 (17)
N2—C24	1.4696 (14)	C19—C20	1.3658 (18)
C1—C2	1.3751 (17)	C19—H19A	0.9300
C1—C6	1.3930 (16)	C20—C21	1.4214 (18)
C2—C3	1.3887 (19)	C20—H20A	0.9300
C2—H2A	0.9300	C21—C22	1.3699 (15)
C3—C4	1.3866 (19)	C21—H21A	0.9300
C3—H3A	0.9300	C22—C23	1.4117 (14)
C4—C5	1.3890 (17)	C22—C24	1.5206 (15)
C4—H4A	0.9300	C25—C26	1.5338 (16)
C5—C6	1.4027 (16)	C25—H25A	0.9700
C5—H5A	0.9300	C25—H25B	0.9700
C6—C7	1.4640 (15)	C26—C27	1.5143 (15)
C7—C8	1.3400 (15)	C26—H26A	0.9800
C7—H7A	0.9300	C27—C32	1.3887 (16)
C8—C12	1.4994 (15)	C27—C28	1.3933 (17)
C8—C9	1.5174 (15)	C28—C29	1.3880 (17)
C9—H9A	0.9700	C28—H28A	0.9300
C9—H9B	0.9700	C29—C30	1.385 (2)
C10—C11	1.5477 (14)	C29—H29A	0.9300
C10—H10A	0.9700	C30—C31	1.381 (2)
C10—H10B	0.9700	C30—H30A	0.9300
C11—C12	1.5210 (15)	C31—C32	1.3863 (18)
C11—C26	1.5510 (14)	C31—H31A	0.9300
C11—C24	1.5628 (15)	C33—H33A	0.9600
C13—C14	1.5110 (15)	C33—H33B	0.9600
C13—C24	1.5998 (14)	C33—H33C	0.9600
			
C13—O2—H1O2	103.9 (17)	C17—C16—H16A	118.8
C10—N1—C9	108.26 (8)	C15—C16—H16A	118.8
C10—N1—C13	103.19 (8)	C16—C17—C18	120.19 (11)
C9—N1—C13	115.37 (8)	C16—C17—H17A	119.9
C33—N2—C25	112.58 (9)	C18—C17—H17A	119.9
C33—N2—C24	115.78 (9)	C23—C18—C19	116.30 (10)
C25—N2—C24	104.66 (9)	C23—C18—C17	116.45 (11)
F1—C1—C2	118.19 (11)	C19—C18—C17	127.24 (11)
F1—C1—C6	117.60 (10)	C20—C19—C18	120.49 (11)
C2—C1—C6	124.20 (11)	C20—C19—H19A	119.8
C1—C2—C3	117.70 (12)	C18—C19—H19A	119.8
C1—C2—H2A	121.1	C19—C20—C21	122.19 (11)
C3—C2—H2A	121.1	C19—C20—H20A	118.9
C4—C3—C2	120.70 (12)	C21—C20—H20A	118.9
C4—C3—H3A	119.6	C22—C21—C20	118.92 (11)
C2—C3—H3A	119.6	C22—C21—H21A	120.5
C3—C4—C5	120.01 (11)	C20—C21—H21A	120.5
C3—C4—H4A	120.0	C21—C22—C23	118.76 (11)
C5—C4—H4A	120.0	C21—C22—C24	132.63 (10)
C4—C5—C6	120.99 (11)	C23—C22—C24	108.61 (9)
C4—C5—H5A	119.5	C18—C23—C14	123.11 (10)
C6—C5—H5A	119.5	C18—C23—C22	123.33 (10)
C1—C6—C5	116.33 (10)	C14—C23—C22	113.55 (10)
C1—C6—C7	119.21 (10)	N2—C24—C22	116.07 (9)
C5—C6—C7	124.33 (10)	N2—C24—C11	102.00 (8)
C8—C7—C6	127.06 (10)	C22—C24—C11	117.98 (9)
C8—C7—H7A	116.5	N2—C24—C13	112.35 (9)
C6—C7—H7A	116.5	C22—C24—C13	104.06 (8)
C7—C8—C12	116.89 (9)	C11—C24—C13	104.01 (8)
C7—C8—C9	124.09 (10)	N2—C25—C26	104.66 (8)
C12—C8—C9	118.60 (9)	N2—C25—H25A	110.8
N1—C9—C8	114.82 (8)	C26—C25—H25A	110.8
N1—C9—H9A	108.6	N2—C25—H25B	110.8
C8—C9—H9A	108.6	C26—C25—H25B	110.8
N1—C9—H9B	108.6	H25A—C25—H25B	108.9
C8—C9—H9B	108.6	C27—C26—C25	113.57 (9)
H9A—C9—H9B	107.5	C27—C26—C11	114.90 (9)
N1—C10—C11	104.03 (8)	C25—C26—C11	103.28 (8)
N1—C10—H10A	110.9	C27—C26—H26A	108.3
C11—C10—H10A	110.9	C25—C26—H26A	108.3
N1—C10—H10B	110.9	C11—C26—H26A	108.3
C11—C10—H10B	110.9	C32—C27—C28	115.55 (10)
H10A—C10—H10B	109.0	C32—C27—C26	120.40 (10)
C12—C11—C10	106.98 (8)	C28—C27—C26	124.03 (10)
C12—C11—C26	115.13 (8)	C29—C28—C27	121.67 (12)
C10—C11—C26	117.17 (9)	C29—C28—H28A	119.2
C12—C11—C24	109.69 (8)	C27—C28—H28A	119.2
C10—C11—C24	101.18 (8)	C30—C29—C28	120.42 (13)
C26—C11—C24	105.62 (8)	C30—C29—H29A	119.8
O1—C12—C8	122.83 (10)	C28—C29—H29A	119.8
O1—C12—C11	123.26 (10)	C31—C30—C29	119.89 (12)
C8—C12—C11	113.85 (8)	C31—C30—H30A	120.1
O2—C13—N1	108.10 (8)	C29—C30—H30A	120.1
O2—C13—C14	111.89 (9)	C30—C31—C32	118.01 (12)
N1—C13—C14	115.09 (9)	C30—C31—H31A	121.0
O2—C13—C24	111.65 (8)	C32—C31—H31A	121.0
N1—C13—C24	105.15 (8)	F2—C32—C31	118.27 (11)
C14—C13—C24	104.78 (8)	F2—C32—C27	117.35 (11)
C15—C14—C23	119.38 (10)	C31—C32—C27	124.38 (12)
C15—C14—C13	131.91 (10)	N2—C33—H33A	109.5
C23—C14—C13	108.69 (9)	N2—C33—H33B	109.5
C14—C15—C16	118.46 (11)	H33A—C33—H33B	109.5
C14—C15—H15A	120.8	N2—C33—H33C	109.5
C16—C15—H15A	120.8	H33A—C33—H33C	109.5
C17—C16—C15	122.38 (11)	H33B—C33—H33C	109.5
			
F1—C1—C2—C3	178.47 (12)	C13—C14—C23—C18	−177.52 (10)
C6—C1—C2—C3	−1.7 (2)	C15—C14—C23—C22	179.49 (10)
C1—C2—C3—C4	−0.5 (2)	C13—C14—C23—C22	0.90 (13)
C2—C3—C4—C5	2.3 (2)	C21—C22—C23—C18	0.68 (17)
C3—C4—C5—C6	−2.1 (2)	C24—C22—C23—C18	−178.72 (10)
F1—C1—C6—C5	−178.26 (10)	C21—C22—C23—C14	−177.73 (11)
C2—C1—C6—C5	1.88 (18)	C24—C22—C23—C14	2.88 (13)
F1—C1—C6—C7	5.77 (16)	C33—N2—C24—C22	−37.58 (13)
C2—C1—C6—C7	−174.09 (12)	C25—N2—C24—C22	86.96 (10)
C4—C5—C6—C1	0.05 (17)	C33—N2—C24—C11	−167.22 (9)
C4—C5—C6—C7	175.79 (11)	C25—N2—C24—C11	−42.68 (10)
C1—C6—C7—C8	−145.45 (13)	C33—N2—C24—C13	81.99 (12)
C5—C6—C7—C8	38.93 (18)	C25—N2—C24—C13	−153.47 (9)
C6—C7—C8—C12	175.81 (11)	C21—C22—C24—N2	−60.40 (17)
C6—C7—C8—C9	3.43 (19)	C23—C22—C24—N2	118.87 (10)
C10—N1—C9—C8	49.53 (12)	C21—C22—C24—C11	61.06 (17)
C13—N1—C9—C8	−65.47 (12)	C23—C22—C24—C11	−119.66 (10)
C7—C8—C9—N1	148.66 (11)	C21—C22—C24—C13	175.62 (13)
C12—C8—C9—N1	−23.59 (14)	C23—C22—C24—C13	−5.10 (11)
C9—N1—C10—C11	−74.36 (10)	C12—C11—C24—N2	150.00 (8)
C13—N1—C10—C11	48.39 (10)	C10—C11—C24—N2	−97.23 (9)
N1—C10—C11—C12	72.81 (10)	C26—C11—C24—N2	25.36 (10)
N1—C10—C11—C26	−156.20 (9)	C12—C11—C24—C22	21.57 (12)
N1—C10—C11—C24	−41.99 (10)	C10—C11—C24—C22	134.33 (9)
C7—C8—C12—O1	27.68 (17)	C26—C11—C24—C22	−103.08 (10)
C9—C8—C12—O1	−159.51 (11)	C12—C11—C24—C13	−93.02 (9)
C7—C8—C12—C11	−149.72 (10)	C10—C11—C24—C13	19.75 (10)
C9—C8—C12—C11	23.09 (14)	C26—C11—C24—C13	142.34 (8)
C10—C11—C12—O1	136.13 (11)	O2—C13—C24—N2	0.39 (12)
C26—C11—C12—O1	4.01 (16)	N1—C13—C24—N2	117.38 (9)
C24—C11—C12—O1	−114.92 (12)	C14—C13—C24—N2	−120.91 (9)
C10—C11—C12—C8	−46.47 (11)	O2—C13—C24—C22	126.74 (9)
C26—C11—C12—C8	−178.59 (9)	N1—C13—C24—C22	−116.27 (9)
C24—C11—C12—C8	62.47 (11)	C14—C13—C24—C22	5.44 (10)
C10—N1—C13—O2	85.18 (9)	O2—C13—C24—C11	−109.14 (9)
C9—N1—C13—O2	−156.95 (8)	N1—C13—C24—C11	7.85 (10)
C10—N1—C13—C14	−148.94 (9)	C14—C13—C24—C11	129.56 (9)
C9—N1—C13—C14	−31.08 (12)	C33—N2—C25—C26	170.68 (10)
C10—N1—C13—C24	−34.20 (10)	C24—N2—C25—C26	44.13 (11)
C9—N1—C13—C24	83.67 (10)	N2—C25—C26—C27	99.05 (10)
O2—C13—C14—C15	56.48 (16)	N2—C25—C26—C11	−26.05 (11)
N1—C13—C14—C15	−67.42 (15)	C12—C11—C26—C27	114.79 (10)
C24—C13—C14—C15	177.62 (12)	C10—C11—C26—C27	−12.32 (13)
O2—C13—C14—C23	−125.16 (10)	C24—C11—C26—C27	−124.03 (10)
N1—C13—C14—C23	110.94 (10)	C12—C11—C26—C25	−120.97 (10)
C24—C13—C14—C23	−4.02 (11)	C10—C11—C26—C25	111.91 (10)
C23—C14—C15—C16	0.81 (17)	C24—C11—C26—C25	0.20 (11)
C13—C14—C15—C16	179.03 (11)	C25—C26—C27—C32	161.80 (10)
C14—C15—C16—C17	−1.58 (19)	C11—C26—C27—C32	−79.59 (13)
C15—C16—C17—C18	0.4 (2)	C25—C26—C27—C28	−16.44 (14)
C16—C17—C18—C23	1.38 (18)	C11—C26—C27—C28	102.17 (12)
C16—C17—C18—C19	−179.11 (12)	C32—C27—C28—C29	−1.88 (17)
C23—C18—C19—C20	−0.13 (18)	C26—C27—C28—C29	176.44 (11)
C17—C18—C19—C20	−179.64 (13)	C27—C28—C29—C30	−0.24 (19)
C18—C19—C20—C21	−0.4 (2)	C28—C29—C30—C31	1.01 (19)
C19—C20—C21—C22	1.1 (2)	C29—C30—C31—C32	0.43 (18)
C20—C21—C22—C23	−1.20 (18)	C30—C31—C32—F2	177.91 (11)
C20—C21—C22—C24	178.01 (12)	C30—C31—C32—C27	−2.81 (19)
C19—C18—C23—C14	178.27 (11)	C28—C27—C32—F2	−177.23 (10)
C17—C18—C23—C14	−2.17 (17)	C26—C27—C32—F2	4.38 (15)
C19—C18—C23—C22	0.01 (17)	C28—C27—C32—C31	3.48 (17)
C17—C18—C23—C22	179.57 (11)	C26—C27—C32—C31	−174.91 (11)
C15—C14—C23—C18	1.08 (17)		

### Hydrogen-bond geometry (Å, °)

**Table:**

Cg1 and Cg2 are the centroids of the C14–C18/C23 and C1–C6 rings, respectively.

**Table:**

*D*—H···*A*	*D*—H	H···*A*	*D*···*A*	*D*—H···*A*
O2—H1O2···N2	0.88 (3)	2.06 (3)	2.6786 (13)	127 (2)
C10—H10A···F1^i^	0.97	2.37	3.3135 (14)	163
C30—H30A···F2^ii^	0.93	2.45	3.1525 (17)	132
C5—H5A···Cg1^iii^	0.93	2.94	3.6110 (14)	131
C33—H33C···Cg2^iv^	0.96	2.93	3.7253 (15)	141

Symmetry codes: (i) *x*, *y*+1, *z*; (ii) −*x*+1, *y*+1/2, −*z*−1/2; (iii) *x*, −*y*−1/2, *z*−3/2; (iv) *x*, −*y*−1/2, *z*−1/2.
